# Polyvinyl alcohol-citrate-stabilized silver nanoparticles as an optical sensor for selective colorimetric determination of sildenafil

**DOI:** 10.55730/1300-0527.3499

**Published:** 2022-09-20

**Authors:** Asiye Aslıhan AVAN

**Affiliations:** Department of Chemistry, Faculty of Engineering, İstanbul University-Cerrahpaşa, İstanbul, Turkey

**Keywords:** Sildenafil, silver nanoparticles, colorimetric detection, sensor, pharmaceutical analysis

## Abstract

A facile and sensitive colorimetric detection method for sildenafil citrate (SLDC) has been developed by using polyvinyl alcohol-citrate-stabilized silver nanoparticles (PVA-Cit-AgNPs). The sensitivity and selectivity of colorimetric detection were investigated in detail. The PVA-Cit-AgNPs could be induced to aggregate immediately in the presence of SLDC, especially after the addition of 0.1 M HCl solution. The SLD could be detected by the colorimetric response of AgNPs that could be monitored by a UV–Vis spectrophotometer or even the naked eye. The linear range was found to be 0.6–5.0 μM with the calculated detection limit of 0.1 μM and the naked-eye detection limit was determined as low as 0.6 μM. The proposed method is successfully employed for the determination of SLDC in commercially available tablets. Meanwhile, the excipients present in the pharmaceuticals do not interfere with the assay procedure.

## 1. Introduction

Sildenafil (SLD) is a pyrazolo[4,3-d]pyrimidin-7-one maintaining a methyl substituent, a propyl substituent, and a 2-ethoxy-5-[(4-methyl piperazine-1-yl) sulfonyl]phenyl group. Viagra is a drug that possesses the dynamic substance sildenafil. The operative element in Viagra, sildenafil, belongs to a group of drugs named phosphodiesterase-type-5 (PDE5) inhibitors. It performs by plugging the PDE5 enzyme, which normally breaks down a fragment known as cyclic guanosine monophosphate [1–4]. Viagra is one of the most counterfeit drugs in the world. Counterfeit Viagra pills themselves can be loaded with harmful elements or materials. Analysis of the ranges of counterfeit PDE5 shows inconsistent doses of active pharmaceutical elements, contaminants (including talcum powder, commercial paint, and printer ink) and alternative elements that are potentially unsafe [5]. Medicines purchased outside the regulated supply chain can be risky. Thus, there is a pressing need to explore an accurate and reliable method for the detection and monitoring of SLD.

A literature review discloses that different analytical methods have been reported up to the present for the quantitative estimation of SLD [6]. Sophisticated techniques, such as electrochemical [7–10], and chromatography [11–19] have been applied to the estimation of SLD in various specimens (pharmaceutical samples, dietary products, herbal products, and biological matrices) [6]. Quantification of trace-level biopharmaceuticals (structurally mimics compounds) in complex biological samples has been of increasing in demand. These samples usually comprise a great number of unknown compounds in addition to the analytes of SLD [14–19]. Therefore, liquid chromatography-mass spectrometry (LC-MS) is one of the paramount techniques for the quantification of trace-level biopharmaceuticals in complex samples [13–17]. However, LC–MS has a high initial cost to buy the instrument which is prohibitive for many laboratories and as a result, the wider utilization of the LC-MS techniques is currently limited. Excepting these techniques, colorimetric method comes as a saviour in the context of saviour time and spending as well. UV-Vis spectrophotometry is a versatile technique and widely utilized for quantitative estimation because of its widespread availability in laboratories, the simplicity of spectrophotometry methods, and its precision and accuracy. To date, several spectrophotometric approaches for the quantitative estimation of SLD have been declared, each detection system has its benefits and drawbacks regarding sensitivity, selectivity, and convenience. Due to the functional organic chromophores carried in the SLD molecule, the UV absorption band of the SLD displays two characteristic bands at 220 and 292 nm, respectively. Thus, a lot of UV-based approaches were employed for the quantitative estimation of SLD [20–22] in a chemically pure form and medicine formulations. Specifically, SLD is an n-electron donor compound and can easily give charge-transfer complexes with σ- or π-acceptors. In this regard, a colorimetric assay method was properly improved for the sensing of sildenafil citrate (SLDC) based on the charge transfer complexation reaction of the SLDC [23]. Also, extractive UV-Vis spectrophotometric methods are reported for the sensing of SLDC. The approaches comprise the formation of colored CHCl_3_ extractable ion-pair complexes with bromocresol green and chromoxane cyanine in an acid environment [24]. Another spectrophotometric method of SLDC utilizing some chromotropic acid-based azo dyes is introduced by Issa et al. [25]. The mentioned methods are based on the ion association of the SLDC with two functional groups (-OH and −SO_3_H) of chromotropic acid azo dyes [25]. In aqueous solutions, SLD exhibits the lowest fluorescence intensity and restricted linear detection range. Nevertheless, in the existence of a cationic and an anionic surfactant, a great fluorescence intensity improvement was monitored by Wang et al. [26,27].

Noble metal nanoparticle (NMNP)-based colorimetric assays are user-friendly and fast detection methods that take benefit of different chemical reaction mechanisms. The universality of NMNPs as sensors or probes is in part due to their highly responsive colorimetric attributes. Aggregation of NMNPs (Au, Ag, Pt, Pd, and so on) due to reaction with target analytes changes the initial color of NMNPs. Among the known NMNPs, gold nanoparticles/silver nanoparticles (AuNPs/AgNPs) have been extensively exploited because of their outstanding optical and electrical attributes [28,29]. NMNPs exhibit outstanding visual attributes in well-dispersed solutions, based on their aggregation level which is mostly established by their specific surface plasmon resonance (SPR) profiles. Furthermore, NMNPs often have extraordinary optical features because they are small enough to limit their electrons and produce quantum efficiency. The red (AuNPs) or yellow (AgNPs) color could be distinguished to the bare eye, even at picomolar levels [30]. This is a key factor for bare-eye sensing implementations because alterations of their surface charge are transformed into a visible color change [31]. More importantly, NMNPs also have a high molar extinction coefficient (ɛ) and this feature is based commonly on their shape, size, and inter-particle distance. Such features enable them to compete with conventional detection systems, such as absorption and fluorescence spectroscopy. Owing to their versatility, adaptability, low cost, and high sensitivity, NMNPs-based protocols have been improved for the sensing of heavy metal ions, small molecules, proteins, enzymes, and so on. AuNPs have been extensively studied for biological and chemical detections as well as colorimetric sensing applications. Although AgNPs have been less used than AuNPs in colorimetric sensing systems, nevertheless, very interesting works have been published regarding sensors based on aggregation, etching and growth of AgNPs [32]. In 2020, two simple and sensitive (fluorimetric and spectrophotometric) methods were improved for the quantitative estimation of SLDC, dapoxetine, vardenafil, and tadalafil using AuNPs [33]. Recently, metal-organic frameworks (MOFs)-AgNPs architectures were fabricated as an efficient surface-enhanced Raman scattering (SERS) substrate. The obtained MOFs-AgNPs substrate provided a route for sensitive SLD and pioglitazone hydrochloride assay. Moreover, the MOFs-AgNPs demonstrate high SERS efficiency than usual AgNPs [34]. For colorimetric approaches, AgNP-based colorimetric methods have some benefits compared with AuNPs. Particularly, AgNP extinction coefficients (~5.56 × 10^8^ M^−1^ cm^−1^ λ = 412 nm) [30] are higher than those of AuNPs (~2.7 × 10^8^ M^−1^ cm^−1^ at λ = 520 nm) [35] of the same average size, on the contrary, AuNP-based colorimetric sensing protocols being reasonably popular. This tendency could be interpreted by the fact that AgNPs functionalization always leads to their chemical deterioration and thereafter, the surface of AgNPs can be easily and rapidly oxidized, thus decreasing their stability.

This study aims to establish a simple, rapid, and inexpensive method for SLD sensing in pharmaceutical specimens at low levels using PVA-Cit-AgNPs and UV-Vis spectroscopy and the naked eye as a detection technique.

## 2. Materials and methods

### 2.1. Materials

During this work, Polyvinyl alcohol (PVA) (Mw 9000–10,000 mol^−1^), AgNO_3_, NaBH_4_, HCl, trisodium citrate (TSC), SLDC, dapoxetine, vardenafil, and tadalafil were used as received without further purification. All reagents were purchased from Sigma-Aldrich. All solutions were prepared using distilled water. The measurements were performed against distilled water blank in a Varian Cary 100 Bio model UV-Vis spectrophotometer (Varian, Inc., USA). All samples were measured from 300 to 800 nm using a UV (quartz) cuvette 500 μL and 10 mm path length. The prepared AgNPs were characterized by Transmission electron microscopy (TEM) (FEI TALOS F200S TEM, USA) and Fourier transform infrared spectroscopy (FTIR) (FTIR Spectrum Two, Perkin Elmer, USA).

### 2.2. Synthesis of AgNPs

AgNPs were prepared according to a previous report with some modifications [36]. AgNPs were easily synthesized based on a chemical reduction protocol involving the reduction of Ag^+^ by NaBH_4_ used as the reducing agent in the presence of TSC and PVA used as a stabilizing agent. This is a fast process at room temperature [37,38]. For AgNP synthesis in darkness, the test tubes used were wrapped completely with aluminium foil. AgNPs can be simply synthesized by AgNO_3_ reduction with NaBH_4_ resulting in the fabrication of unbalanced AgNPs the reason for this is the generation of NaOH. However, when PVA was added as a stabilizer, it resulted in the fabrication of steady AgNPs [39]. In a typical experiment, 1.5 mL of 1.0% AgNO_3_ (w/v), 10 mL of 2% TSC (w/v), and 2.0 mL of 0.1% PVA (w/v) were added to a 100 mL round bottom flask and adjusted volume to 100 mL with distilled water. Subsequently, the mixture was stirred (10 min) and cooled in an ice bath. Zero temperature was picked for synthesis because at normal temperature, the AgNPs were grown more speedily, and aggregation occurred, resulting in the fabrication of unbalanced AgNPs. Then, 2 mL of NaBH_4_ (0.1% w/v) was joined dropwise to the mentioned solution under magnetic stirring. Meanwhile, the clear colorless solution gives a bright yellow color indicating the fabrication of AgNPs. After that, the colored solution was further stirred at zero temperature for 2 h, kept in a brown bottle, and stored at 4 °C [39,40]. The obtained AgNPs were denoted PVA-Cit-AgNPs.

In this system, the citrate is loosely adsorbed to the core of AgNPs and thereby stabilizes the AgNPs colloids by charge repulsion. Meanwhile, polymer molecule (PVA) adsorbs onto the AgNP surface through multiple noncovalent interactions and it offers a steric repulsion effect to ensure greater stability. Besides, the PVA avoid the metal nanoparticles from coming close to each other and the stabilization is more effective in an aqueous solution [36]. The optimum condition for synthesizing homogenous AgNPs as follows: 1.5 mL of 1.0% AgNO_3_ (w/v), 10 mL of 2% TSC (w/v), 2.0 mL of 0.1% PVA (w/v), and 2 mL 0.1% NaBH_4_ (w/v).

### 2.3. Recommended procedure for the determination of sildenafil

Colorimetric and naked-eye detection was carried out using PVA-Cit-AgNPs solution. For detection of SLD, 0.1 mL HCl (1 M) and various concentrations of SLD (0.6–5.0 μM) were mixed and then diluted to 0.7 mL with distilled water. After that, the PVA-Cit-AgNPs solutions (0.3 mL) were added quickly and the mixture was shaken well. The prepared solutions were allowed to stand for 15 min in the dark at normal temperature and the changes occurring in each tube were observed visually or photographed in color. The magnitude of the absorbance ratio (A_575_/A_408_) was carefully recorded. In this sensing system, the order of reagent addition is a key parameter.

### 2.4. Assay of pharmaceutical formulation

All pharmaceutical formulations or samples were obtained from regional markets (İstanbul/Turkey). The contents of two kinds of pharmaceutical tablets (25 mg and 50 mg) were determined by the suggested method. Four SLD tablets were crushed and powdered. An exactly weighed part of the powder equivalent to 25 or 50 mg of SLD was transferred into a 50 mL volumetric flask. Ten mL of 0.050 M HCl was joined into the flask and shaken well. Next, the flask was filled to the mark with the distilled water and mixed, then filtered through a Whatman No. 42 filter paper. Solutions of working range concentration (0.6–5.0 μM) were prepared by proper dilution of the stock sample solution with distilled water and used for the analysis. Analysis was performed as described in the recommended procedure for the determination of sildenafil.

### 2.5. Method optimization

All the experimental variables that affect the absorbance intensity were tested to reach the maximum sensitivity. Several factors that may affect the reaction were studied; these factors included PVA-Cit-AgNPs concentration and volume, HCl concentration and volume, PVA concentration, order of addition of the colorimetric reagent and reaction time. For that purpose, solutions containing 3.0 μM of SLDC were prepared and measured under different conditions. The intensity of absorbance ratio (A_575_/A_408_) was recorded and used.

### 2.6. Method validation

The invented method was validated according to the International Council for Harmonization of Technical Requirements for Pharmaceuticals for Human Use (ICH) guidelines. In this sense, the different validation parameters were carefully examined, including linearity, range, accuracy, precision, the limit of detection (LOD), the limit of quantitation (LOQ), and specificity.

## 3. Results and discussion

### 3.1. Characterization of sensor

**Figure 1:**
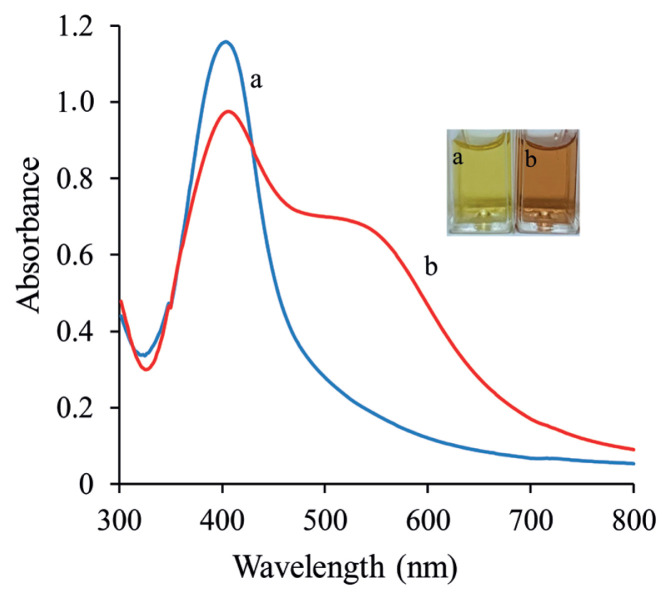
UV–Vis spectra (a and b) and photographs (a, b) of and PVA-Cit-AgNPs in absence and presence of 3 μM SLDC. Reaction conditions: blank; distilled water, HCl; 0.1 M, PVA-Cit-AgNPs: 0.3 mL.

Figure 1 shows the absorption spectra and the color change of PVA-Cit-AgNPs aqueous in the absence (a) and presence (b) of SLD. A decrease of maximum absorption intensity at 408 nm and an enhancement of a new absorption peak at 575 nm point out that the situation of the AgNPs changed from dispersion to aggregation form. In the presence of SLD, the color of AgNPs changed from yellow to red (Figure 1 photographs a and b) defining the aggregation of the AgNPs, which is following the result of UV-Vis spectroscopy. Therefore, the AgNPs show a higher sensitivity to SLD. Simultaneously, a new broad peak (located around 575 nm) was found due to the interaction between PVA-Cit-AgNPs and the SLD molecules in the solution. To gain further information about the features of the sensor was studied by other common approaches such as FTIR and TEM analysis.

### 3.2. FTIR spectra and TEM image of PVA-Cit-AgNPs

**Figure 2:**
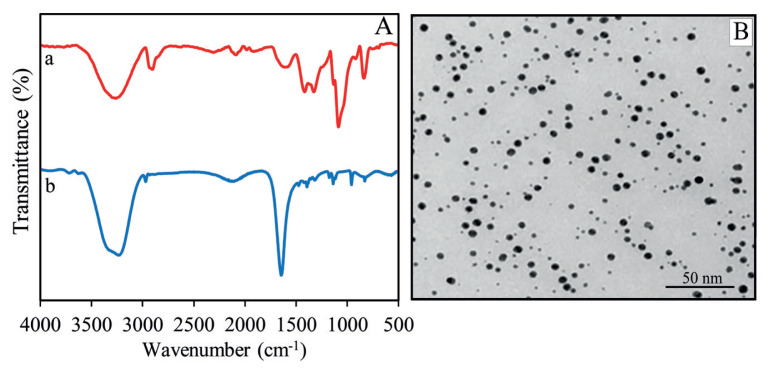
(A) FTIR spectra of pure PVA (a) and PVA-Cit-AgNPs (b), (B) TEM image of PVA-Cit-AgNPs.

Figure 2A (spectrum a) shows the FTIR spectrum of pure PVA. A vigorous vibration peak belonging to the −OH peak has appeared at 3250 cm^−1^. The characteristic peaks at 1617 cm^−1^ (−C–C vibrations), at 1088 cm^−1^ (−C–O vibrations), at 922 cm^−1^ (−C–O stretching), and at 840 cm^−1^ (−C–H bending vibrations). On the other hand, PVA-Cit-capped AgNPs yielded new absorption peaks at 3230 cm^−1^ (symmetric stretching of carboxylate anion), at 1636 cm^−1^ (O–H bending), at 1471 cm^−1^ (C–H bending), and 943 cm^−1^ (C–O stretching). In addition, small peaks were also observed at 2977 cm^−1^ and 2897 cm^−1^, related to the stretching of −CH_2_– (asymmetric and symmetric stretching, respectively). As a result, all of the prepared AgNPs were thoroughly decorated by their capping agents [41]. FTIR spectrum of PVA capped AgNPs is shown in Figure 2A (spectrum b). The spectrum pattern of PVA-Cit-AgNPs displayed shifting in the peaks due to the interaction of PVA with the surface of Cit-AgNPs by chemical adsorption. Hence, we can verify that prepared AgNPs are successfully captured by Cit and PVA [42].

TEM study of PVA-Cit-AgNPs: AgNPs formed in PVA supports verified using TEM (Figure 2B). The shape and size of the PVA-Cit-AgNPs were calculated from the TEM profile. This fabrication route and reaction conditions lead to the generation of nonagglomerated AgNPs of 11–14 nm in size and the TEM profile revealed spherical-shaped AgNPs.

### 3.3. Effect of presence and absence of PVA on AgNPs preparation

**Figure 3:**
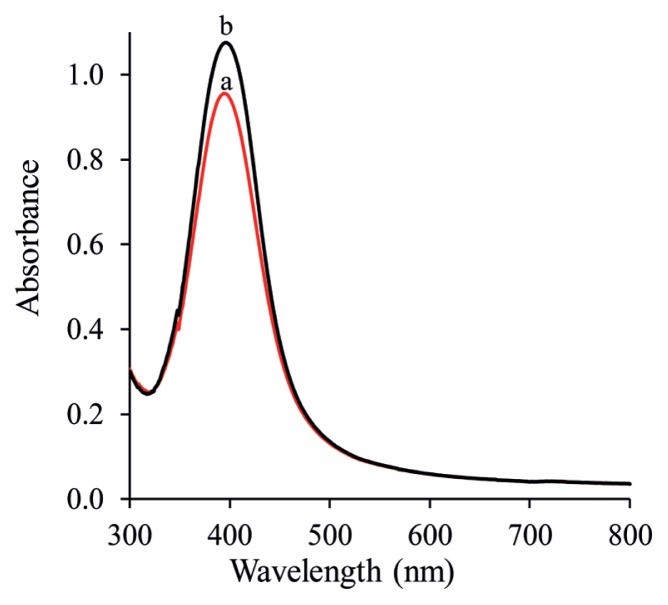
Absorbance spectrum of Cit-AgNPs (a) and PVA-Cit-AgNPs (b).

Figure 3 displays the UV-Vis profile of the AgNPs synthesized using Cit and NaBH_4_ as a reducing agent and with the presence and absence of PVA. The results reveal the surface plasmon resonance peaks around 408 nm for all systems, which is typical for AgNPs. The wavelength of maximum absorbance is a characteristic value, designated as λ_max_ = 408 nm. On the other hand, the intensities of maximum absorbance are both the same as can be seen in Figure 3. The maximum absorption peak of AgNPs and PVA-Cit-AgNPs were both located at 408 nm, illustrating that PVA polymer had little impact on the optical images. These reflections explained above could be attributed to the reduction process of silver ions to AgNPs using NaBH_4_ followed by binding between AgNPs at the cluster surface, while the polymeric chain defends the cluster from blending with the next silver ion. The obtained zeta potential of the Cit-AgNPs (close to zero value) is higher than that of the PVA-citrate-AgNPs (−17.3 ± 0.8 mV vs. −48.4 ± 0.8 mV), and theoretically, they should be more prone to aggregation. Furthermore, the value of the zeta potential gives knowledge about AgNP stability. The higher the value of potential exhibits increased electrostatic repulsion and therefore increased nanoparticle stability. Therefore, PVA-Cit-AgNPs had higher particle stability than Cit-AgNPs.

### 3.4. Colorimetric sensing mechanism of SLD

**Figure 4:**
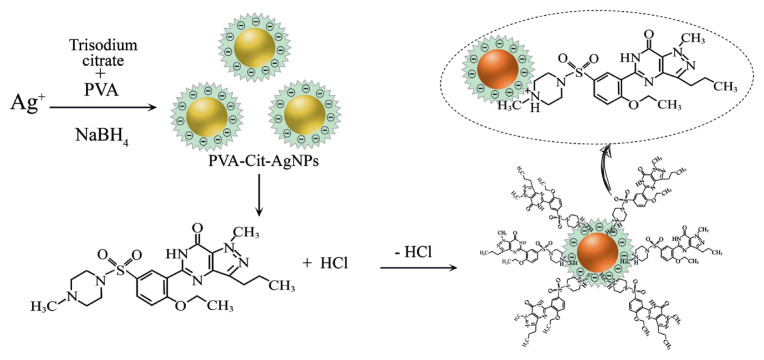
Probably reactions mechanism of AgNPs with SLD.

Sildenafil molecule has three reactive functional groups, namely piperazine, pyrimidinone, and pyrazole [24]. SLD is a positively charged molecule [43]. As expected, due to the presence of citrate as a stabilizer, the AgNPs are negatively charged and the reached zeta potential was −48.4 ± 0.8 mV. In this case, the negatively charged AgNPs pull some of the positive ions (counter ions) in the dispersion [44]. As sildenafil citrate molecules contain groups of different natures like pyrimidine and pyrazole and piperazine. Pyrimidine and pyrazole groups are fused heterocycles, their protonations are difficult due to the combination of steric and resonance effects. Therefore, one NH group of the piperazine ring is merely protonated and can act as a hydrogen-bond donor. Consequently, anionic AgNPs form ion-pair bindings with the positively (cationic) charged groups. The negative charge confers electrostatic attraction between AgNPs and positively charged SLDC of the molecules, thereby facilitating AgNP attachment onto SLDC molecules. When SLDC is introduced with these functionalized AgNPs, then it interacts with piperazine groups (with protonated N atom). Due to this interactivity, aggregation of AgNPs occurs. Considering, PVA-Cit-AgNPs a suitable probe was used for carrying out colorimetric determinations of SLD in pharmaceutical samples. Based on the above discussion, the probable mechanism of formation of the complex is shown in Figure 4.

### 3.5. Effect of HCl concentration

**Figure 5:**
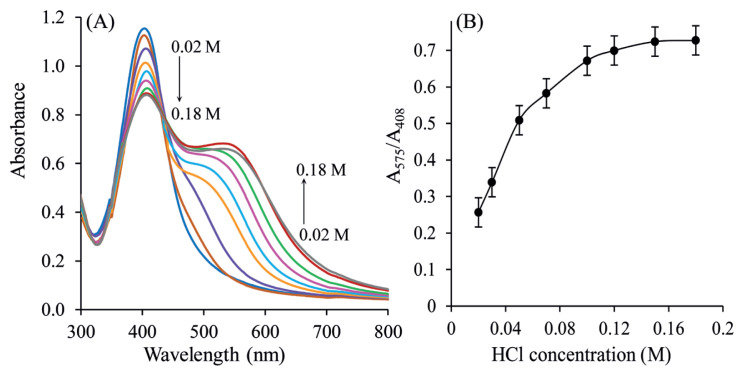
(A) Effect of HCl concentration on the UV*-*Visible absorbance of PVA-Cit-AgNPs-SLDC (B) variation of absorbance *(*A_575_/A_408_) with different HCl concentrations (0.02, 0.03, 0.05, 0.07, 0.10, 0.12, 0.15, and 0.18 M). SLDC: 3 μM, reaction time: 15 min, blank: distilled water.

The highest response at PVA-Cit-AgNPs was obtained in HCl solutions. The influence of HCl on the detection of the SLDC with the aggregation of PVA-AgNPs was manifested by the value of A_575_/A_408_ shown in Figure 5. The effect of the HCl concentration on the absorbance was studied in the range 0.02–0.18 M HCl and the reached results are presented in Figure 5. The absorbance change increased by increasing the HCl concentration up to 0.1 M and decreased at higher concentrations. Increasing HCl concentration caused a decrease in absorbance change. Therefore, 0.1 M HCl was used as the optimum concentration. The higher the concentration of HCl used during the determination, the color of the sample changes from yellow to dark red or rusty-brown and finally a precipitate is obtained. This means that AgNPs can react with HCl to form AgCl, showing unusually high chemical reactivity toward HCl [45]. In this case, the absorbance of SLDC in the solution cannot be measured accurately. Therefore, excessive use of HCl should be avoided.

### 3.6. Effect of PVA concentration

**Figure 6:**
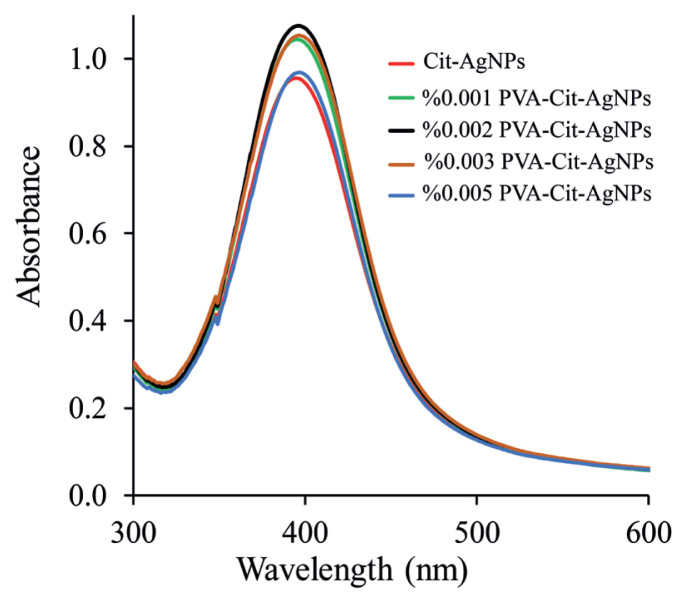
UV-Vis spectra of AgNPs prepared using PVA at different concentrations (0.001%–0.005% (w/v)).

Surface modification of AgNPs with a stabilizing agent, such as PVA, plays a crucial role in shape-controlled seeded growth and colloidal stability. The polymer PVA was used as a shape and size controlling agent and stabilizing agent [46]. The polymer PVA acts may assemble on a nanoparticle inorganic core so that it can regulate nanoparticle morphology and form-factor (growth and shape). Therefore, the length and flexibility of PVA chains can administer its adsorption feature on a nanoparticle surface. In this regard, different concentrations of PVA ranging from 0.001% to 0.005% (w/v) were investigated. The UV-Vis spectra of the AgNPs were obtained using TSC as a reducing agent and with the presence and absence of PVA which functions as a stabilizing agent. The formation of PVA stabilized AgNPs was confirmed by UV–Vis spectroscopy by the appearance of a peak at 408 nm. Figure 6 shows the UV–Vis spectra of AgNPs prepared using PVA at different concentrations (0.001%–0.005% (w/v)). It is also clear that there is a gradual increase in the absorption intensity, by increasing the PVA concentration up to 0.002% (w/v) which could be attributed to the increment in the stabilization performance of the formed AgNPs. In presence of PVA, the reduction process was provided completely and a sharp absorption band already appeared at 408 nm. Over this concentration value, the reduction performance diminished because the presence of PVA is formed. In this case, the absorption spectra may symbolize the high viscose of PVA that would behave as a barrier between silver ions and the NaBH_4_ thereby reducing the AgNPs production. Therefore, in this approach, 2.0 mL of 0.1% PVA (final volume 100 mL) was used in the experiment.

### 3.7. Effect of reaction time

**Figure 7:**
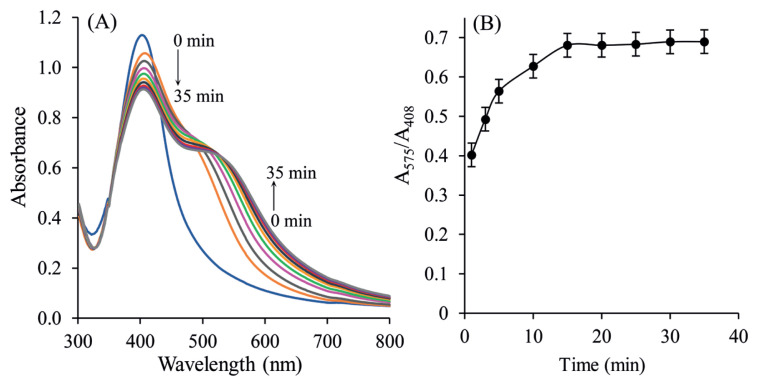
(A) UV-Vis spectra of PVA-Cit-AgNPs-SLDC with different reaction times (1, 3, 5, 10, 15, 20, 25, 30, and 35 min). SLDC: 3 μM and HCl: 0.1 M. (B) Variation of absorbance *(*A_575_/A_408_) with different reaction times.

The reaction time played a critical role in the sensing unit. Therefore, the reaction time on the colorimetric response was also tested. Figure 7 shows that a longer reaction time led to higher responses for the detection of SLDC. The absorbance ratio of SLDC increased rapidly within 10 min, changed confidential, and then remained almost stable when the duration exceeded 15 min. After 15 min, the UV-Vis response tended to not increase any further, indicating that the PVA-Cit-AgNPs-SLDC interaction reached saturation. For this reason, 15 min reaction time was chosen for further experiments. Accordingly, the longer the incubation time in the analyte solution, the lower the colorimetric signals that were recorded, with a levelling off for reaction times longer than 35 min. Due to collapse (turbidity), a longer reaction period would have false consequences.

### 3.8. Analytical performance

**Figure 8:**
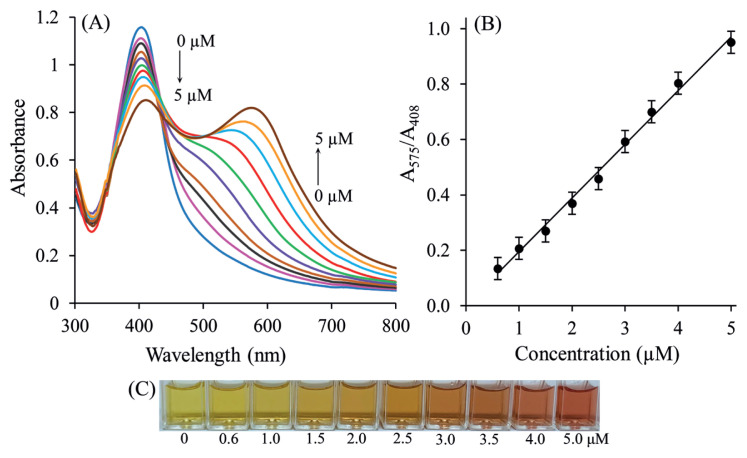
(A) UV-Vis spectra of PVA-Cit-AgNPs as a function of SLDC concentrations. Figure (B) shows the linear calibration curve between absorbance intensity ratio A_575_/A_408_ and the concentrations of SLDC. (C) Photographs of PVA-Cit-AgNPs as a function of SLDC concentrations.

The UV–Vis absorption spectra of AgNPs with different concentrations of SLDC were depicted under optimal sensing conditions. With increasing of SLDC concentration from 0.6 to 5.0 μM, the absorption peak at 408 nm progressively decreases, and the UV–Vis absorption peak at 575 nm increases progressively, which displays that more and more AgNPs aggregate. Figure 8A shows the UV–Vis absorption spectra of AgNP-based sensors reacted with various concentrations of SLDC (0.6–5.0 μM). Figure 8B exhibits a good linear relationship between A_575_/A_408_ and SLDC levels in the range of 0.6–5.0 μM. The photographic profile of AgNP-based sensors reacted with various concentrations of SLD is shown in Figure 8C. On the basis of the test results, the regression equation of colorimetric sensor was: A_575_/A_408_ = 0.1938 C (μM) + 0.0011 (R^2^ = 0.9943). Consequently, the ratio of the absorbance values at (A_575_/A_408_) was linear in the SLDC concentration range of 0.6–5.0 μM (or 0.40–3.33 μg/mL) (Figure 8B). The limits of detection (LOD) and limit of quantification (LOQ) values of SLC were calculated as 0.1 μM (or 0.06 μg/mL) and 0.4 μM (0.26 μg/mL), respectively. These values were calculated on using following equations: LOD = 3Sb/m; LOQ = 10Sb/m, where Sb is the standard deviation of three measurements of the reagent blank, and m, the slope of the calibration graph. Furthermore, with naked eye detection, the lowest possible concentration of SLDC was found to be 0.6 μM (or 0.4 μg/mL) (Figure 8C). These findings indicate that AgNP-based visual sensors can be used for the quantitative analysis of SLDC. The precision (repeatability) of the colorimetric sensor, expressed as RSD, was carefully checked on five individual specimens comprising 3 μM of SLDC and obtained to be 2.8%.

### 3.9. Comparison with other spectrophotometric methods

The as-prepared PVA-Cit-AgNPs sensor also exhibits comparative sensitivity and selectivity compared with previously reported UV-Vis spectrophotometric methods (Table 1) [22–25,33]. From the comparison as illustrated in Table 1, it established that the improved sensing unit offered a sufficient linear detection range and low LOD value. Also, the use of PVA makes this sensing unit eco-friendly, selective, and cost-effective. The comparison results indicated that the as-prepared PVA-Cit-AgNPs are convenient for the detection and quantification of SLDC.

### 3.10. Interference study

Under the optimum condition of spectrophotometry, potential interfering species including Mg^2+^, Ca^2+^, Ti^3+^, Al^3+^, PO_4_^3−^ SO_4_^2−^ NO_3_^−^ and lactose were examined individually on the sensor responses for sensing of SLDC at 3 μM concentration. The effect of each potential interfering species was tested up to a ratio (analyte: interferent ratio) of 1:10 mol/mol, being monitored with no considerable changes in analytical peaks resolution and peak intensities of SLDC. Furthermore, due to the amine functional groups, the colorimetric determination of SLC with PVA-Cit-AgNPs is interfered with by dapoxetine, vardenafil, and tadalafil (1:1), in acidic solution at A_575_/A_408_, significantly.

### 3.11. Analytical application in pharmaceutical samples

Having investigated the selectivity and potential implementation of this suggested method with pure SLDC solutions, the declared sensing strategy was examined by handling a commercially available pharmaceutical sample, that is, in the presence of other interfering species. To validate the colorimetric strategy, a recovery protocol was carried out with two symbolic pharmaceuticals from those displayed in Table 2. Satisfactory average recoveries for both samples (99% for 25 mg and 97% for 50 mg SLD) were obtained, which approves the practicality and accuracy of the sensing strategy for SLD quantification in commercial tablets. A comparison of the reached consequences of the AgNP-based strategy with those of the UV-Vis (λ = 220 nm) method [21] demonstrated acceptable agreement between both protocols. The obtained UV-Vis results are tabulated in Table 2. According to the results, the improved method can be used without hesitation for the rapid and sensitive detection of SLD in pharmaceutical samples. More importantly, to the active ingredient, sildenafil citrate (viagra), each commercial drug contains the following inactive ingredients: titanium dioxide, magnesium stearate, dibasic calcium phosphate, lactose, microcrystalline cellulose, anhydrous croscarmellose sodium, hypromellose, triacetin, and FD & C Blue #2 aluminium lake. The additives in the tablet have no interference effect. The results (shown in Table 2) agree with the manufacturers’ stated contents of SLD.

## 4. Conclusion

In this examination, a simple colorimetric method to perform the SLDC determination in pharmaceutical samples using UV-Vis spectroscopy has been developed. This protocol is based on the distance-dependent visual behaviours of AgNPs. The assay explained in this work is detectable with the naked eye. The colorimetric sensor has a limit of detection of 0.1 μM which is to the best of our knowledge the lowest ever reported for the colorimetric detection of SLDC using PVA-Cit-AgNPs. Furthermore, a noteworthy feature of this detection method is that it is a simple technique displaying high selectivity and sensitivity to SLDC over other examined ions and excipients.

## Figures and Tables

**Table 1:** Comparison of the developed colorimetric method with some recent spectrophotometric methods.

Spectrophotometric method	Linear range (μg/mL)	LOD (μg/mL)	Reference
UV-Vis	10–50	NR	[21]
FIA-UV-detection	0.66–3.33	0.20	[22]
Utility of certain σ and π-acceptors	10–260	1.5	[23]
Extractive method	1.25–25	0.18	[24]
Ion-associate formation methods	0.8–125	0.30–2.70	[25]
AuNPs based method	0.2–1.0 0.002–0.12^a^	0.06 0.006^a^	[33]
AgNPs based method	0.40–3.33	0.06	This method

a Fluorometric method

**Table 2:** Determination of SLD in real pharmaceutical tablets by AgNPs-based colorimetric method (n = 3).

Sample	Taken (μg)	Found UV-Vis (μg ± SD)	Added (μg)	Found (μg ± SD)	Recovery (%)
1	0.58	0.59 ± 0.03	-	0.58 ± 0.04	-
			0.28	0.86 ± 0.02	98
			0.57	1.15 ± 0.03	100
			0.85	1.43 ± 0.05	100
2	0.50	0.49 ± 0.01	-	0.49 ± 0.04	-
			0.24	0.73 ± 0.01	98
			0.45	0.94 ± 0.05	98
			0.72	1.19 ± 0.02	97
